# Influence of alcohol on the worsening of COVID-19 and the occurrence of long COVID^1^

**DOI:** 10.1590/1518-8345.7679.4813

**Published:** 2026-03-30

**Authors:** Mirella Machado Ortiz Modesto, Natan Nascimento de Oliveira, Natan David Pereira, Wanessa Cristina Baccon, Lígia Carreira, Maria Aparecida Salci

**Affiliations:** 1 Universidade Estadual de Maringá, Maringá, PR, Brazil.; 2 Scholarship holder at the Coordenação de Aperfeiçoamento de Pessoal de Nível Superior (CAPES), Brazil.

**Keywords:** Alcohol Drinking, COVID-19, Syndemic, Post-Acute COVID-19 Syndrome, Adult, Aged.

## Abstract

**Objective:**

to analyze the alcohol consumption patterns of adults and older adults before the development of COVID-19 and the influence of alcohol consumption on the outcomes and complications of long COVID.

**Method:**

cross-sectional study based on data from a retrospective cohort conducted with adults and older adult who had COVID-19 and who consumed alcohol before infection with the disease. A standardized electronic form was used to collect sample data and a path model was adjusted to prove the theoretical model on the influence of alcohol consumption on negative outcomes for COVID-19.

**Results:**

sample of 1,171 participants who responded to the question about alcohol. Of these, 408 (34.84%) reported alcohol consumption prior to the disease. The majority were male, younger, highly educated, and had children over the age of 18. The presence of chronic noncommunicable diseases leads to an 11% increase in the chance of hospitalization and a 12% increase in the chance of long COVID. The age of the participants affected alcohol use and directly affected the need for hospitalization.

**Conclusion:**

It is important to adopt intervention strategies aimed at reducing alcohol consumption, especially in contexts of syndemic, to mitigate the associated risks.

## Highlights

**(1)** Those who consumed alcohol prior to COVID-19 had a higher risk of hospitalization. **(2)** Alcohol consumption was linked to hospitalization in long COVID. **(3)** Most participants who consumed alcohol had a preexisting condition.

## Introduction

COVID-19 has triggered an unprecedented health crisis, contributing to the widening of social inequalities among different populations around the world and establishing different ways of dealing with a new social routine due to political, cultural, and economic changes^1^.

Because of this, several authors^2^ ^-^ ^4^ have pointed out the syndemic characteristics of COVID-19, because in addition to having global repercussions, it has also produced synergistic effects caused by the interaction of COVID-19 with other existing diseases, which have increased the incidence and potentiated the social effects^5^ ^-^ ^6^. 

Several public health interventions occurred during the pandemic, including social distancing and the closure of bars, restaurants, and nightclubs. This social behavior fostered a considerable increase in alcohol consumption and the adoption of alcohol abuse behaviors in private spaces/homes, added to pre-existing vulnerabilities, resulting in high levels of stress, causing increased tension in family relationships and a greater risk of developing mental disorders and various forms of suffering^7^ ^-^ ^9^.

In this context, it is important to highlight that alcohol consumption is related to social, behavioral, psychological, and economic factors. These factors express the pattern of alcoholic beverage consumption among the Brazilian and global populations^10^.

In addition, chronic alcohol consumption impairs the immune system, making the body susceptible to viral and bacterial infections, with a major impact on severe respiratory infections such as COVID-19^11^.

In a study of 416 adults in the United States, 62.9% reported increased alcohol consumption during the pandemic, with a 14.9% increase in heavy drinking (p<0.001) and a 65% increase in alcohol abuse (p=0.0439). In addition to easy access to alcohol (76%)^12^. Another study, also in the US, revealed a 20% increase in alcohol consumption patterns during the pandemic^13^.

People who have developed COVID-19 or have a history of probable infection have also been prone to long-term symptoms, better known as post-acute sequelae of SARS-CoV-2 infection (PASC) or “Long COVID”. The symptoms of long COVID are diverse and include fatigue, dyspnea, mental confusion, memory and concentration problems, chest and joint pain, and multiple organ dysfunction. They may reappear after initial recovery from the acute phase of COVID-19 or persist since the first occurrence of the disease^14^.

Long COVID usually occurs three months after the onset of COVID-19, with symptoms lasting at least two months. Currently, there are no proven treatments for long COVID, and it cannot be explained by an alternative diagnosis^14^ ^-^ ^15^. 

It should be noted that, in contexts affected by COVID-19, the interaction with other diseases, mainly chronic noncommunicable diseases (NCDs), driven by political, economic, and social factors, has been characterized as a syndemic^2^.

Thus, producing new knowledge, deepening the understanding of the experience of alcohol consumption before and after COVID-19, the changes that occurred in individual and family lifestyles during this period, as well as the strategies used to maintain physical health and reduce diseases and health problems, becomes imperative to science^16^.

Given this scenario, we aim to answer the following research question: did alcohol consumption by adults and the older adult before COVID-19 aggravate the disease and influence the occurrence of long COVID? In this sense, the objective of the study was to analyze the pattern of alcohol consumption among adults and the older adult who developed COVID-19 and the influence of alcohol consumption on the outcomes and complications of long COVID.

## Method

### Study design

This is a retrospective cross-sectional study based on data from a retrospective cohort conducted with adults and older adult who had COVID-19 in the state of Paraná^17^. The study followed the recommendations of STrengthening the Reporting of OBservational studies in Epidemiology (STROBE) in its construction.

### Setting

This study was part of a larger project called “Coorte COVID-19 Paraná/UEM” (Paraná/UEM COVID-19 Cohort), developed in southern Brazil, specifically in the state of Paraná, and uses data sharing as a methodological strategy. The main objective of the project was to investigate the short-, medium-, and long-term repercussions of COVID-19 on adults and older adult living in the state of Paraná. The research was conducted throughout the state, which covers 399 municipalities organized into 22 Health Regions and four Macroregions: East, West, North, and Northwest. As of December 31, 2020, Paraná had recorded 413,412 confirmed cases of COVID-19 and 7,912 deaths from the disease^18^.

### Period

The project data were collected between March 11, 2020, and December 31, 2020, after a minimum period of 12 months from the notification or hospital discharge of confirmed cases of COVID-19 by SARS-CoV-2, as recorded in the notification systems. However, sample data collection began in April 2021 and ended in March 2022, with each interview lasting at least 50 minutes.

### Population

Participants in this study included adults aged between 18 and 59 years old, as well as older adult aged 60 years and older, who had been diagnosed with COVID-19 or long COVID and who resided in the state of Parana.

### Selection criteria

The inclusion criteria for this study were: being over 18 years of age; having a confirmed diagnosis of COVID-19 or long COVID. Regarding exclusion criteria, observations in which the answers to the questions “Before COVID-19, did you drink alcohol?” and “Long COVID?” were null were removed, as were pregnant women, postpartum women, and participants who died for any reason between the notification period and the application of the questionnaire.

### Definition of the sample or participants

Based on the mandatory notification forms for Severe Acute Respiratory Syndrome associated with Coronavirus (SARS-CoV) in the State of Paraná, registered in the Influenza Syndrome Epidemiological Surveillance System (Sivep Gripe), a system accessible to all epidemiological surveillance centers for monitoring and controlling reported severe cases, 1,190 potential participants were selected using the stratified sampling method described in the larger study^17^, 1,190 potential participants were selected from among adults and older adult people who were diagnosed with COVID-19 or long COVID and received care in public and/or private health services between March 11, 2020, and December 31, 2020.

After the selection criteria, the study ended up with 1,171 observations, in which the participants were divided into three distinct subgroups: those who received outpatient care 312 (41%), those who were hospitalized in wards 220 (29%), and those who required ICU admission 231 (30%), both in the Unified Health System (SUS) and in private institutions in the state of Paraná.

### Study variables

The occurrence of long COVID (no and yes), alcohol use before COVID-19 (no and yes), and hospitalization for COVID-19 treatment (no and yes) were listed as dependent variables. The primary outcome was the occurrence of long COVID, while alcohol and hospitalization were considered secondary outcomes and mediators of long COVID.

The independent variables were the sociodemographic and clinical characteristics of the participants, namely: gender (female and male), age (mean), age group (adult or older adult), race/color (white and black, brown, Asian, and indigenous), education (up to 7 years of schooling and 8 years or more), marital status (with partner and without partner), number of children under 18 (no and yes), chronic disease (no and yes), and place of treatment for acute COVID-19 (outpatient clinic, ward, and ICU).

### Instruments used for gathering information

The data source for the larger study was the notification records of the State *Notifica COVID-19* System (*Notifica COVID-19*) of the state of Paraná, and for cases requiring hospitalization (ward and intensive care unit (ICU), the study participants were identified and selected from the mandatory notification forms for Severe Acute Respiratory Syndrome (SARS) associated with SARS-CoV-2, entered into the Influenza Syndrome Epidemiological Surveillance System (SIVEP-Gripe).

The instrument used for data collection in the study was a standardized electronic form completed by members (nurses and graduate students in the health field) of the “COVID-19 Paraná/UEM Cohort” project. The instrument consists of four main thematic blocks, classified as follows: (I) Initial identification of the participant and interviewer; (II) Cohort history (subdivided into block (II.1) Signs and Symptoms of COVID and Post-COVID Syndromes; (II.2) History of COVID-19 and Post-COVID-19; (II.3) Personal History); (III) Sociodemographic, Occupational, and Economic/Financial Characterization of the participant; and (IV) Final interview information. 

The question about alcohol consumption patterns before COVID-19 is part of block II.3 Personal History. Participants were asked if they drink/drank alcohol, and for those who answered yes, they were asked how many days a week they consumed alcohol (1 to 7 days), what types of drinks (beer; wine; martini/vermouth; cachaça/vodka/whiskey), and the approximate daily amount in milliliters (ml). 

The WHO values in effect during the period^19^ ^)^ were used as the parameter for Brazilian alcoholic units, which were later updated, maintaining the same calculation formula, based on slightly different dosages. For the WHO, a standard dose contains 10 grams (g) or 12.7 milliliters (mL) of pure ethanol. This dose is equivalent to 285 mL of regular beer, 100 mL of wine, or 30 mL of spirits^20^.

### Data collection

The interviews were conducted via recorded telephone calls, lasting at least 50 minutes, using an electronic form as a guide. Data collection was conducted by nurses and graduate students in the health field, who received at least 20 hours of training to conduct the research.

After data collection, the Informed Consent Form (ICF) was sent to participants in PDF format, via online platforms or email. It was decided to conduct interviews by telephone or online to overcome geographical limitations, allowing the participation of patients in all regions of the state of Paraná, and also to follow social distancing recommendations.

The collected data were stored in a database that allowed only one form submission per IP (Internet Protocol), in order to ensure the security of the information obtained.

Personal and/or sensitive information that allowed for the identification and contact of participants was obtained in advance from individual registration forms, combining three databases: the Brazilian COVID-19 epidemiological surveillance database, the Influenza Syndrome Epidemiological Surveillance System (Sivep Gripe) database, and the Paraná State COVID-19 Notification System, known as *Notifica COVID-19 Paraná*. Databases 1 and 2 cover cases of hospitalization for Severe Acute Respiratory Syndrome (SARS) due to COVID-19 (severe and moderate cases), while database 3 covers outpatient or mild cases of the disease.

### Data processing and analysis

**Figure 1:**
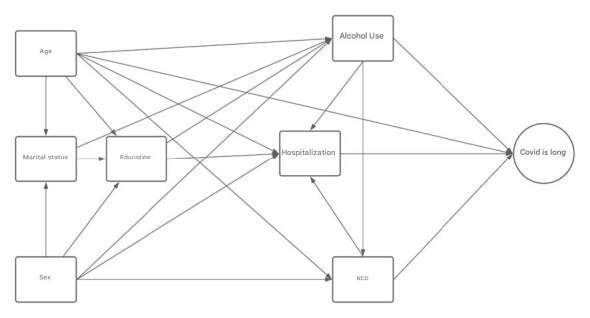
Theoretical model of the influence of alcohol on COVID-19 outcomes

The data were tabulated in spreadsheets and analyzed using R software, version 4.3.0. A descriptive analysis of the variables was performed by estimating the absolute and relative frequencies of the categorical variables, as well as the mean and standard deviation of the numerical variables. Pearson’s chi-square test and Mann-Whitney test were used to identify differences between groups. To understand the complex relationships between alcohol use and COVID-19, Structural Equation Models (SEM)^21^ were adjusted, specifically through path analysis. This multivariate technique was employed because it allows testing hypothesized theoretical models while estimating the direct and indirect relationships between multiple variables. This approach is particularly suitable for investigating multifactorial phenomena, such as the proposed interactions between COVID-19, alcohol use, and social determinants of health. Exogenous variables (only causal) were considered, i.e., those that influence other variables within the model without being influenced by any of them within the scope of this study; this group included age, gender, marital status, and education level. Endogenous variables (caused by other variables/mediators) are those that are influenced by one or more variables within the model. Each endogenous variable was modeled as an outcome for one or more predictor variables, namely: Alcohol use before COVID-19, Chronic Noncommunicable Disease (CNCD), Hospitalization for COVID-19 treatment, and, finally, Long COVID, the latter representing the main final outcome that was modeled, being influenced by all the previous variables, directly and/or indirectly (Figure 1). This model took into account the concept of COVID-19 as a syndemic^2^ of alcohol use disorders and social determinants of health. The proposed relationships were then quantified using estimated coefficients in the form of odds ratios. This structural equation modeling technique was based on covariance (CB-SEM) and logit structure, using the lavaan package in R. As analysis criteria, all variables underwent verification for multicollinearity (VIF), Chi-Square, Comparative Fit Index (CFI), Loglikelihood, and Root Mean Square Error of Approximation (RMSEA).

*NCDs = Non-Communicable Chronic Diseases

### Ethical aspects

The ethical precepts of Resolutions No. 466/2012 and No. 510/2016 of the National Health Council (CNS) (Brazil, 2012) were complied with. The research project “COVID-19 Paraná/UEM Cohort” has been approved by the Permanent Research Ethics Committee with Human Beings (COPEP) of the State University of Maringá (UEM), with opinion number 4.165.272/2020 and with Certificate of Ethical Review (CAAE) number 34787020.0.0000.0104 issued on July 21, 2020. 

The participants involved in data collection were informed about the objectives and method used in the research, as well as information about voluntary participation, availability, and willingness to participate in the research. The right of access to data was ensured. 

Free and informed consent and permission for the interviews to be recorded were obtained orally at the time of telephone contact with the interviewees. The ICF was then sent to participants electronically or by mail.

## Results

A total of 1,171 study participants who answered questions regarding alcohol use were selected. Of these, 763 (65.16%) indicated that they did not use alcohol prior to COVID-19, while 408 (34.84%) reported alcohol consumption before the disease (Table 1). 

**Table 1:** Characteristics of study participants who reported alcohol use prior to COVID-19 infection. Maringá, PR, Brazil, 2024

Variables	Alcohol use before COVID-19			
	No (N = 763)	Yes (N = 408)	Test	p-value
**Gender**			QQ*	<0.001
Female	440 (58%)	121 (30%)		
Male	323 (42%)	287 (70%)		
**Age**			KW^†^	<0.001
Mean (Standard deviation)	55.3 (16.0)	50.5 (15.4)		
**Age group**			QQ*	<0.001
Adult	381 (50%)	260 (64%)		
Older adult	382 (50%)	148 (36%)		
**Race/Colour**			QQ*	0.990
White	494 (65%)	264 (65%)		
Black/Brown/Yellow/Indigenous	269 (35%)	144 (35%)		
**Education**			QQ*	<0.001
Up to 7 years of study	310 (41%)	104 (25%)		
8 years or more of study	453 (59%)	304 (75%)		
**Marital status**			QQ*	0.844
With partner	513 (67%)	272 (67%)		
No partner	250 (33%)	136 (33%)		
**Children under 18 years of age**			QQ*	0.009
No	655 (86%)	326 (80%)		
Yes	108 (14%)	82 (20%)		
**Chronic disease**			QQ*	0.055
No	258 (34%)	161 (39%)		
Yes	505 (66%)	247 (61%)		
**Acute COVID treatment center**			QQ*	0.386
Outpatient clinic	312 (41%)	150 (37%)		
Infirmary	220 (29%)	125 (31%)		
ICU	231 (30%)	133 (33%)		
**Long COVID**			QQ*	0.336
No	251 (33%)	123 (30%)		
Yes	512 (67%)	285 (70%)		

*QQ = Pearson’s chi-square test; ^†^KW = Kruskal-Wallis test

Among participants who reported alcohol consumption, there was a higher prevalence of males, younger ages, higher education levels, and children over the age of 18. 

### Path analysis

A path model was adjusted to test the theoretical model listed above regarding the influence of alcohol consumption on negative COVID-19 outcomes, such as hospitalization and long COVID.

**Figure 2:**
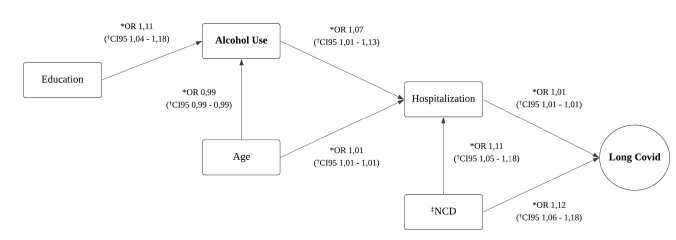
Path analysis of the impact of alcohol consumption on COVID-19 outcomes

In Figure 2, it can be seen that alcohol use did not directly influence the onset of long COVID; on the other hand, its consumption was associated with hospitalization, being directly related to long COVID.

Path analysis revealed significant associations between different variables studied. Alcohol use showed a positive association with hospitalization for COVID-19, in which, for each unit increase in the measure of alcohol use, the chance of being hospitalized for COVID-19 increased by about 7%. On the other hand, age showed a negative association with alcohol use, indicating that with increasing age, the chance of using alcohol decreases by about 1%. Education was positively associated with alcohol use, showing an increase of about 11% in the chance of alcohol use as the level of education increases. The presence of NCDs in the patient leads to an 11% increase in the chance of hospitalization and a 12% increase in the chance of long COVID.

Similarly, it was found that the age of participants affected alcohol use, with the greatest influence on those closer to adulthood, as well as directly affecting the need for hospitalization. Furthermore, education was associated with alcohol use, as already mentioned.

*OR = Odds Ratio; ^†^CI = Confidence Interval; ^‡^NCD = Chronic noncommunicable diseases

## Discussion

The sociodemographic profile of the sample studied emphasized the conditions of alcohol consumption patterns. Most participants did not consume alcohol before contracting COVID-19. In another previous study, most participants (59.7%) reported a reduction in consumption since social distancing measures were implemented^8^. 

The decrease in alcohol use may have been driven by reduced access to alcoholic beverages and decreased monthly income to purchase them. Many individuals lost their jobs during social isolation at the onset of COVID-19, which resulted in reduced or ceased family income and, in many cases, economic and food insecurity, as evidenced by other studies^22^ ^-^ ^23^. 

The economic instability generated during the COVID-19 pandemic has resulted in increased poverty and social vulnerability^24^. Therefore, treating COVID-19 as a syndemic means considering it in an integrated manner, also taking into account the social and economic factors of the affected population^25^.

Alcohol consumption before COVID-19 was associated with men. The study, conducted as part of a national cross-sectional survey in Slovenia to record the number of alcoholic beverages consumed during the pandemic compared to the year before COVID-19, showed changes in consumption in the first survey phase among younger adults, men, and people with higher education. This can be explained by the flexibility of marketing measures, restaurants, hotels, and shopping centers at the beginning of the survey^26^.

Another study conducted in Berlin, whose overall objective was to generate representative data on pandemic-related stress, parental stress, the subjective and mental health of parents, and the occurrence of adverse childhood experiences (ACEs), showed that parents were stressed by the closure of schools (55.9%), the closure of daycare centers (52.1%), and restrictions on outdoor activities (46.1%). In addition, parental stress was significantly higher than before COVID-19. Parents in this sample reported symptoms of anxiety and depression, and 37% of parents said they had abused alcohol or drugs at home throughout their children’s lives, with 5.1% saying the problems had worsened significantly during the pandemic (11.3%)^27^.

This study showed, through the pathway model, that alcohol consumption did not directly influence the onset of long COVID, but that its consumption was associated with hospitalization, which is directly related to long COVID. 

In this hierarchical association, alcohol use can be considered a risk factor for prolonging the disease in individuals infected with COVID-19, as well as for a potential worsening of cases, leading to more hospitalizations^28^. 

Alcohol has a detrimental effect on the immune system, impairs the mucociliary barrier of the respiratory tract, and reduces the innate response and resistance to the virus. This increases the chance of pulmonary complications after SARS-CoV-2^29^ and the likelihood of hospitalization^30^.

In this study, people who consumed alcohol before COVID-19 were more likely to require hospitalization, either in a ward or, in severe cases, in the ICU. Although viral load is an important risk factor, it is understood that, in isolation, levels or patterns of alcohol consumption increase the risk of harmful consequences not only in physical dimensions, but also mental and social ones for those who consume it^28^. 

Regarding the chronic diseases analyzed, it was observed that most participants who had previously consumed alcohol had some preexisting disease. 

In a study conducted with a sample of 821 men diagnosed with substance use disorder, who were admitted to a specialized drug addiction unit at a public hospital in Porto Alegre, Brazil, it was found that 305 were alcohol users, 233 cocaine/crack users, and 283 men who used multiple substances (including alcohol). Of these, alcohol users had a higher number of risk factors for more severe forms of COVID-19 compared to the other groups. Alcohol use was related to metabolic and liver diseases, as well as the prevalence of hypertension (26.6% to 38%), heart disease (6.7% to 11.5%), and cirrhosis (4.4% to 7.9%)^28^. 

It should be noted that existing physical health vulnerabilities may also reflect on overall health risk conditions. Thus, considering the prevalence of adults and older adults who consumed alcohol before COVID-19, there are risk factors for COVID-19 severity^29^, which represent a higher chance of hospitalization and may be related to the manifestation of long COVID.

An online cohort study aimed at determining sociodemographic factors found that lifestyle, medical history prior to COVID-19, and characteristics of SARS-CoV-2 infection were associated with long COVID. The same study also showed that the average number of participants who consumed alcohol prior to COVID-19 was 4.19±5.71, and of these, 47.7% still reported symptoms of long COVID at the time of the study^12^. Another analysis highlighted that adult participants with high rates of alcohol use (27.3%) had symptoms of long COVID^31^.

Furthermore, the possible consequences of consumption in people who are vulnerable due to underlying diseases may have had a substantial impact on the quality of life of the interviewees, as well as having a negative impact on their health, since alcoholic beverages directly or indirectly affect the immune system, as pointed out in a previous study^32^.

In view of the above, it is important that future studies consider the socioeconomic specificities associated with alcohol consumption and the severity pattern associated with COVID-19 and long COVID, in order to expand the evidence and contribute to health promotion and prevention strategies among the adult population and, especially, the older adult, since they may have chronic diseases directly associated with the severity of long COVID. 

Reflecting on the concept of syndemic^2^ allows us to construct the dimension between biological issues, for the development of a disease, and social issues, which favor them, thus highlighting how these factors interact with each other at both the population and individual levels to aggravate the burden of disease^33^.

The limitations of the study mainly refer to the bias in the responses, which may have been influenced by the recollections of participants, family members, and/or caregivers, as well as the fact that the data were self-reported or even left unanswered. This may lead to bias in our estimate of people who consumed alcohol before COVID-19. 

However, despite the limitations mentioned, it is important to highlight that the study was conducted with data from an important cohort in the state of Parana, which had a significant number of participants and was a pioneer in the use of this method in the state during the pandemic period. Furthermore, it should be emphasized that several tests were performed to minimize the risk of response bias and loss of data that were relevant to understanding the epidemiological scenario related to COVID-19 and long COVID in the state of Paraná.

The study contributes to the advancement of scientific knowledge by highlighting the complex interaction between syndemic, alcohol consumption, and COVID-19, especially in adults and the older adult. By integrating multiple dimensions (biological, social, and behavioral), the research contributes to more integrated and intersectoral approaches in the planning of public health actions.

In the field of nursing, research strengthens the understanding of syndemic as an essential concept for qualifying clinical practice, health surveillance, and the formulation of educational and preventive interventions. In addition to highlighting the strategic role of nursing in health promotion and comprehensive care in complex contexts.

## Conclusion

In this cross-sectional assessment, based on data from a retrospective cohort study conducted with adults and older adult who had COVID-19 in the state of Paraná, it was found that alcohol use did not directly influence the onset of long COVID, but its consumption was associated with hospitalization, being directly related to long COVID. In addition, the age group of the participants affected alcohol consumption, with the greatest influences on those closer to adulthood, as well as directly affecting the need for hospitalization.

Furthermore, alcohol use was more associated with males and those with higher levels of education, showing an increase of about 11% in the chance of alcohol use as the level of education increases. The presence of NCDs in patients leads to an 11% increase in the chance of hospitalization and a 12% increase in the chance of long COVID.

Therefore, this study recommends practical measures, such as screening protocols for alcohol consumption in post-COVID-19 patients, especially in primary care, as well as public health education campaigns on the risks of alcohol in health crises, focusing on vulnerable groups (such as men and individuals with higher education) and professional training are important to integrate the alcohol agenda into the management of patients with COVID-19 and long COVID. 

## Data Availability

Datasets related to this article will be available upon request to the corresponding author.
