# Supplementary material for: Influence of alcohol on the worsening of COVID-19 and the occurrence of long COVID
Source: Rev Lat Am Enfermagem. 2026 Mar 30;34:e4813. doi: 10.1590/1518-8345.7679.4813 (PMC13035203; doi:10.1590/1518-8345.7679.4813)
Supplement: Translation

# Translation: Influência do álcool no agravamento da COVID-19 e na ocorrência de COVID longa^2^

**DOI:** 10.1590/1518-8345.7679.4814

**Authors:** Mirella Machado Ortiz Modesto, Natan Nascimento de Oliveira, Natan David Pereira, Wanessa Cristina Baccon, Lígia Carreira, Maria Aparecida Salci

**Affiliations:** 1 Universidade Estadual de Maringá, Maringá, PR, Brasil.; 2 Bolsista da Coordenação de Aperfeiçoamento de Pessoal de Nível Superior (CAPES), Brasil.

**Keywords:** Consumo de Bebida Alcoólica, COVID-19, Sindemia, Síndrome Pós-COVID-19 Aguda, Adulto, Idoso.

## Abstract

**Objetivo:**

analisar o padrão de consumo de álcool de adultos e idosos antes do desenvolvimento da COVID-19 e a influência do consumo de álcool nos desfechos e agravos da COVID longa.

**Método:**

estudo transversal ancorado em dados de uma coorte retrospectiva conduzida com adultos e idosos que tiveram COVID-19 e que consumiram álcool antes da infecção pela doença. Utilizado formulário eletrônico padronizado para coleta de dados da amostra e ajustado modelo de caminhos, para comprovar o modelo teórico sobre a influência do consumo de álcool em desfechos negativos para COVID-19.

**Resultados:**

amostra com 1.171 participantes respondentes à questão do álcool. Destes, 408 (34,84%) relataram o consumo de álcool antes da doença. Prevaleceu o sexo masculino, com idades mais baixas, escolaridade elevada e com filhos maiores de 18 anos. A presença de Doenças Crônicas Não Transmissíveis acarreta um aumento de 11% na chance de hospitalização e 12% na chance de COVID de longa duração. A idade dos participantes afetou o uso de álcool e afetou diretamente a necessidade de hospitalização.

**Conclusão:**

é importante adotar estratégias de intervenções voltadas para a redução do consumo de álcool, sobretudo em contextos de sindemia, a fim de mitigar os riscos associados.

## Destaques

**(1)** Quem ingeriu álcool antes da COVID-19 teve maior risco de hospitalização. **(2)** O consumo de álcool foi relacionado à hospitalização na COVID longa. **(3)** A maioria dos participantes que consumiam álcool tinham alguma doença preexistente.

## Introdução

A COVID-19 desencadeou uma inestimável crise sanitária, contribuindo para a ampliação das desigualdades sociais entre diversas populações do mundo e instituiu diferentes modos de lidar com uma nova rotina social, em razão das alterações políticas, culturais e econômicas^1^_._

Em virtude desse fato, diversos autores^2^ ^-^ ^4^ ^)^ apontaram as características sindêmicas da COVID-19, pois além de ter produzido repercussões a nível global, ela também, produziu efeitos sinérgicos causados pela interação da COVID-19 com outras doenças já existentes, que aumentaram a incidência e potencializaram os efeitos sociais^5^ ^-^ ^6^_._

Diversas intervenções de saúde pública ocorreram durante a pandemia, incluindo o distanciamento social e a proibição do funcionamento de bares, restaurantes e casas noturnas. Esse comportamento social fomentou um considerável aumento do consumo de álcool e adoção de comportamentos de abuso etílico em espaços privados/domicílio, somado a vulnerabilidades pré-existentes, resultando em altos níveis de estresse, ocasionando aumento de tensão nas relações familiares e maior risco de desenvolvimento de transtornos mentais e sofrimentos diversos^7^ ^-^ ^9^_._

Nesse contexto, é importante destacar que o consumo de álcool tem relação com fatores sociais, comportamentais, psicológicos e econômicos. Esses fatores expressam o padrão de consumo de bebidas alcoólicas da população brasileira e mundial^10^_._

Além disso, o consumo crônico de álcool prejudica o sistema imunológico, tornando o organismo suscetível a infecções virais e bacterianas, com grande impacto sobre infecções respiratórias graves, como a COVID-19^11^.

Em um estudo realizado com 416 adultos dos Estados Unidos da América (EUA), 62,9% pessoas relataram o aumento do consumo de álcool durante a pandemia, com 14,9% a mais em consumo pesado (p<0,001) e aumento de 65% em abuso de álcool (p=0,0439). Além da facilidade de acesso ao álcool (76%)^12^. Outra pesquisa, também nos EUA, revelou aumento de 20% no padrão de consumo de bebida alcoólica no período da pandemia^13^.

Pessoas que desenvolveram a COVID-19 ou com histórico de provável infecção, também estiveram sujeitas a apresentarem sintomas de longo prazo, mais conhecidos como sequelas pós-agudas da SARS - Infecção por CoV-2 (PASC) ou “*Long-COVID*”. Os sintomas para COVID longa são diversos e incluem fadiga, dispneia, confusão mental, problemas de memória e concentração, dores no peito e nas articulações, e disfunção de múltiplos órgãos e podem surgir novamente após a recuperação inicial da fase aguda da COVID-19 ou persistir desde a primeira ocorrência da doença^14^.

A condição de COVID longa ocorre, geralmente, três meses após o início da COVID-19, com sintomas que duram pelo menos dois meses. Atualmente, não existem tratamentos comprovados para COVID longa e não podem ser explicados por um diagnóstico alternativo^14^ ^-^ ^15^. 

Ressalta-se que, em contextos afetados pela COVID-19, a interação com outras doenças, principalmente, doenças crônicas não transmissíveis (DCNT), impulsionados por fatores políticos, econômicos e sociais, caracterizou-se como sindemia^2^_._

Assim, produzir novos conhecimentos, aprofundando a compreensão da vivência do processo do consumo de bebida alcoólica antes e após a COVID-19, das mudanças ocorridas no estilo/modo de vida individual e familiar durante este período, bem como as estratégias utilizadas para manutenção da saúde física, diminuição de doenças e agravos à saúde torna-se imperativo à ciência^16^_._

Diante desse cenário, pretende-se responder à seguinte questão de pesquisa: o consumo de bebida alcoólica, por adultos e idosos, antes da COVID-19 agravou o quadro da doença e exerceu influência na ocorrência da COVID longa? Nesse sentido, o objetivo do estudo foi analisar o padrão de consumo de álcool de adultos e idosos que desenvolveram a COVID-19 e a influência do consumo de álcool nos desfechos e agravos da COVID longa.

## Método

### Delineamento do estudo

Trata-se de um estudo transversal retrospectivo, ancorado em dados de uma coorte retrospectiva conduzida com adultos e idosos que tiveram COVID-19 no estado do Paraná^17^. O estudo seguiu as recomendações do *STrengthening the Reporting of OBservational studies in Epidemiology* (STROBE) em sua construção.

### Local

O presente estudo integrou um projeto maior denominado “Coorte COVID-19 Paraná/UEM”, desenvolvido na região Sul do Brasil, especificamente no estado do Paraná, e utiliza o compartilhamento de dados como estratégia metodológica. O objetivo principal do projeto foi investigar as repercussões de curto, médio e longo prazo da COVID-19 em adultos e idosos residentes no estado do Paraná. A pesquisa foi conduzida em todo o estado, o qual abrange 399 municípios organizados em 22 Regionais de Saúde e quatro Macrorregionais: Leste, Oeste, Norte e Noroeste. Até 31 de dezembro de 2020, o Paraná registrou 413.412 casos confirmados de COVID-19 e 7.912 óbitos pela doença^18^.

### Período

Os dados do projeto foram coletados entre 11 de março de 2020 e 31 de dezembro de 2020, após um período mínimo de 12 meses a partir da notificação ou alta hospitalar de casos confirmados de COVID-19 por SARS-CoV-2, conforme registrados nos sistemas de notificação. Porém, a coleta dos dados da amostra teve início em abril de 2021 e finalizou em março de 2022, com duração mínima de 50 minutos cada entrevista.

### População

Participaram deste estudo: adultos, com idade entre 18 e 59 anos completos, bem como idosos a partir de 60 anos, que foram diagnosticados com COVID-19 ou COVID longa, que residiam no estado do Paraná.

### Critérios de seleção

Os critérios de inclusão para este estudo foram: ter idade superior a 18 anos; ter diagnóstico confirmado de COVID-19 ou COVID longa. Sobre os critérios de exclusão, foram removidas as observações em que as respostas das questões “Antes da COVID-19, tomava alguma bebida alcoólica?” e “COVID Longa?” eram nulas, bem como removidas as gestantes, puérperas e participantes que tenham evoluído a óbito por qualquer motivo entre o período da notificação e aplicação do questionário.

### Definição da amostra ou participantes

A partir das fichas de notificação compulsória obrigatória da Síndrome Respiratória Aguda Grave associada ao Coronavírus (SARS-CoV) no Estado do Paraná, registradas no Sistema de Vigilância Epidemiológica de Síndrome Gripal (Sivep Gripe), sistema que é acessível por todos os núcleos de vigilância epidemiológica para monitoramento e controle dos casos graves notificados, foram selecionados, por método de amostragem estratificada descrito no estudo maior^17^, 1.190 potenciais participantes, entre adultos e idosos que foram diagnosticados com COVID-19 ou COVID longa e receberam atendimento em serviços de saúde pública e/ou privados no período entre 11 de março de 2020 e 31 de dezembro de 2020.

Após os critérios de seleção, o estudo finaliza com 1.171 observações, em que os participantes foram distribuídos em três subgrupos distintos: aqueles que receberam atendimento ambulatorial 312 (41%), os que foram hospitalizados em enfermarias 220 (29%) e os que necessitaram de internação em UTI 231 (30%), tanto no Sistema Único de Saúde (SUS) quanto em instituições privadas no estado do Paraná.

### Variáveis do estudo

Foram elencadas como variáveis dependentes a ocorrência da COVID longa (não e sim) o uso de álcool antes da COVID-19 (não e sim) e a hospitalização para o tratamento da COVID-19 (não e sim). O desfecho primário foi a ocorrência da COVID longa, enquanto o álcool e a hospitalização foram considerados como desfechos secundários e mediadores da COVID longa.

Como variáveis independentes, foram elencadas características sociodemográficas e clínicas dos participantes, nomeadamente: sexo (feminino e masculino), idade (média), faixa etária (adulto ou idoso), raça/cor (branca e preto, pardo, amarelo e indígena), escolaridade (até 7 anos de estudo e 8 anos ou mais), situação conjugal (com parceiro e sem parceiro), número de filhos menores de 18 anos (não e sim), doença crônica (não e sim) e local de tratamento da COVID-19 aguda (ambulatório, enfermaria e UTI).

### Instrumentos utilizados para a coleta de informações

Como fonte de dados para o estudo maior, utilizou-se os registros de notificação do Sistema Estadual Notifica COVID-19 (Notifica COVID-19) do estado do Paraná e, para os casos com internação hospitalar (Enfermaria e Unidade de Terapia Intensiva (UTI), os participantes do estudo foram identificados e selecionados a partir das fichas de notificação compulsória da Síndrome Respiratória Aguda Grave (SRAG) associada ao SARS-CoV-2, inseridas no Sistema de Vigilância Epidemiológica de Síndrome Gripal (SIVEP-Gripe).

O instrumento utilizado para coleta de dados da pesquisa foi um formulário eletrônico padronizado, realizado pelos membros (enfermeiros e alunos de pós-graduação na área da saúde) integrantes do projeto “Coorte COVID-19 Paraná/UEM”. O instrumento é composto por quatro blocos temáticos principais, classificados da seguinte forma: (I) Identificação inicial do(a) participante e entrevistador(a); (II) Histórico da Coorte (subdividido no bloco (II.1) Sinais e Sintomas das Síndromes COVID e Pós-COVID; (II.2) Histórico da COVID-19 e Pós-COVID-19; (II.3) Histórico Pessoal; (III) Caracterização Sociodemográfica, Laboral e Econômica/Financeira do(a) participante e (IV) Informações finais da entrevista.

A questão sobre o padrão de consumo de bebida alcoólica antes da COVID-19 faz parte do bloco II.3 Histórico Pessoal. O participante foi questionado se toma/tomava alguma bebida alcoólica e, para os casos de registros positivos, verificava-se quantos dias por semana era feito esse consumo (1 a 7 dias), quais os tipos de bebida (cerveja; vinho; martini/vermute; pinga/vodka/uísque) e a quantidade aproximada diária, em mililitros (ml). 

Empregou-se, como parâmetro das unidades alcoólicas brasileiras, os valores da OMS vigentes no período^19^, os quais passaram por atualização mais tarde, mantendo a mesma fórmula de cálculo, com base em dosagens ligeiramente diferentes. Para a OMS, uma dose padrão contém 10 gramas (g), ou 12,7 mililitros (mL) de etanol puro. Essa dose equivale a 285 mL de uma cerveja comum, 100 mL de vinho ou 30 mL de destilados^20^.

### Coleta de dados

As entrevistas foram realizadas por meio de ligações telefônicas gravadas, com duração mínima de 50 minutos, utilizando um formulário eletrônico como guia. A coleta de dados foi conduzida por enfermeiros e alunos de pós-graduação na área da saúde, os quais receberam treinamento de pelo menos 20 horas para a realização da pesquisa.

Após a coleta de dados, o Termo de Consentimento Livre e Esclarecido (TCLE) foi enviado aos participantes em formato PDF, por meio de plataformas *online* ou por e-mail. Optou-se por realizar entrevistas por telefone ou online para superar limitações geográficas, permitindo a participação de pacientes em todas as regiões do estado do Paraná, e, também, para seguir as recomendações de distanciamento social.

Os dados coletados foram armazenados em um banco de dados que permitia apenas um envio do formulário por IP (Protocolo de Internet), visando garantir a segurança das informações obtidas.

As informações pessoais e/ou sensíveis que permitiam a identificação e o contato com os participantes foram obtidas previamente das fichas de registro individual, combinando três bases de dados: a base de vigilância epidemiológica da COVID-19 do Brasil, a base do Sistema de Vigilância Epidemiológica de Síndrome Gripal (Sivep Gripe), e o Sistema Estadual de Notificação da COVID-19 do Paraná, conhecido como Notifica COVID-19 Paraná. As bases 1 e 2 abrangem casos de internação por Síndrome Respiratória Aguda Grave (SRAG) devido à COVID-19 (casos graves e moderados), enquanto a base 3 engloba casos ambulatoriais ou leves da doença.

### Tratamento e análise dos dados

Os dados foram tabulados em planilhas eletrônicas e analisados por meio do *software* R, versão 4.3.0. Fez-se uma análise descritiva das variáveis, por meio da estimação de frequências absolutas e relativas das variáveis categóricas, além de média e desvio-padrão das variáveis numéricas. Os testes Qui-quadrado de Pearson e o teste de Mann-Whitney foram utilizados para identificação de diferenças entre grupos. Para compreender as relações complexas entre o uso de álcool e a COVID-19, foram ajustados Modelos de Equações Estruturais (MEE)^21^, especificamente por meio da análise de caminhos (*Path Analysis*). Essa técnica multivariada foi empregada pois permite testar modelos teóricos hipotetizados ao mesmo tempo em que estimam as relações diretas e indiretas entre múltiplas variáveis. Essa abordagem é particularmente adequada para investigar fenômenos multifatoriais, como as interações propostas entre COVID-19, uso de álcool e determinantes sociais de saúde. Foram consideradas variáveis exógenas (somente causadoras), isto é, aquelas que influenciam outras variáveis dentro do modelo sem serem influenciadas por nenhuma delas no escopo deste estudo; neste grupo, incluíram-se Idade, Sexo, Estado Civil e Escolaridade. Já as variáveis endógenas (causadas por outras variáveis/mediadoras) são aquelas que são influenciadas por uma ou mais variáveis dentro do modelo. Cada variável endógena foi modelada como um desfecho para uma ou mais variáveis preditoras, sendo elas: Uso de álcool antes da COVID-19, Doença Crônica Não Transmissível (DCNT), Hospitalização para o tratamento da COVID-19 e, por fim, COVID Longa, esta última representando o desfecho final principal que foi modelado, sendo influenciada por todas as variáveis anteriores, direta e/ou indiretamente (Figura 1). Este modelo levou em consideração o conceito da COVID-19 como sindemia^2^ dos transtornos relacionados com o uso de álcool e os determinantes sociais de saúde. As relações propostas foram, então, quantificadas por meio dos coeficientes estimados, na forma de *Odds Ratios*. Esta técnica de modelagem por equações estruturais foi baseada em covariância (CB-SEM) e estrutura *logit*, utilizando o pacote *lavaan* no R. Como critérios de análise, todas as variáveis passaram pela verificação de multicolinearidade (VIF), Qui-Quadrado, *Comparative Fit Index* (CFI), *Loglikelihood* e o *Root Mean Square Error of Approxim*ation (RMSEA).

**Figura 1:** Modelo teórico da influência do álcool sobre os desfechos da COVID-19

*DCNT = Doenças Crônicas Não Transmissíveis

### Aspectos éticos

Foram obedecidos os preceitos éticos das Resoluções n.º 466/2012 e n.º 510/2016, do Conselho Nacional de Saúde (CNS) (Brasil, 2012). O projeto de pesquisa “Coorte COVID-19 Paraná/UEM” está aprovado pelo Comitê Permanente de Ética em Pesquisa com Seres Humanos (COPEP) da Universidade Estadual de Maringá (UEM), com parecer de número 4.165.272/2020 e com Certificado de Apresentação de Apreciação Ética (CAAE) número 34787020.0.0000.0104 emitido em 21 de julho de 2020.

Os participantes envolvidos na coleta de dados foram esclarecidos quanto aos objetivos e ao método utilizado na pesquisa, bem como quanto às informações sobre a participação voluntária, disponibilidade e vontade de participar da pesquisa. Foi assegurado o direito de acesso aos dados. 

O consentimento livre e esclarecido e a permissão para as entrevistas serem gravadas foram obtidas oralmente no momento do contato telefônico com os entrevistados. Em seguida, o TCLE era enviado aos participantes por meios eletrônicos ou via correio.

## Resultados

Foram selecionados 1.171 participantes no estudo que responderam às questões referentes ao uso de álcool. Destes, 763 (65,16%) indicaram não utilizar álcool previamente à COVID-19, enquanto 408 (34,84%) relataram o consumo de álcool antes da doença (Tabela 1). 

**Tabela 1:** Características dos participantes do estudo que relataram o uso de álcool antes da infecção por COVID-19. Maringá, PR, Brasil, 2024

Variáveis	Uso de álcool antes da COVID-19			
	Não (N = 763)	Sim (N = 408)	Teste	valor-p
**Sexo**			QQ*	<0,001
Feminino	440 (58%)	121 (30%)		
Masculino	323 (42%)	287 (70%)		
**Idade**			KW^†^	<0,001
Média (Desvio-padrão)	55,3 (16.0)	50,5 (15.4)		
**Faixa etária**			QQ*	<0,001
Adulto	381 (50%)	260 (64%)		
Idoso	382 (50%)	148 (36%)		
**Raça/Cor**			QQ*	0,990
Branco	494 (65%)	264 (65%)		
Preto/Pardo/Amarelo/Indígena	269 (35%)	144 (35%)		
**Escolaridade**			QQ*	<0,001
Até 7 anos de estudo	310 (41%)	104 (25%)		
8 anos ou mais de estudo	453 (59%)	304 (75%)		
**Situação conjugal**			QQ*	0,844
Com parceiro	513 (67%)	272 (67%)		
Sem parceiro	250 (33%)	136 (33%)		
**Filhos menores de 18 anos**			QQ*	0,009
Não	655 (86%)	326 (80%)		
Sim	108 (14%)	82 (20%)		
**Doença crônica**			QQ*	0,055
Não	258 (34%)	161 (39%)		
Sim	505 (66%)	247 (61%)		
**Local de tratamento COVID aguda**			QQ*	0,386
Ambulatório	312 (41%)	150 (37%)		
Enfermaria	220 (29%)	125 (31%)		
UTI	231 (30%)	133 (33%)		
**COVID longa**			QQ*	0,336
Não	251 (33%)	123 (30%)		
Sim	512 (67%)	285 (70%)		

*QQ = Teste Qui-quadrado de Pearson; ^†^KW = Teste de Kruskal-Wallis

Dentre os participantes que relataram o consumo de álcool, percebe-se maior prevalência do sexo masculino, com idades mais baixas, escolaridade elevada e com filhos maiores de 18 anos. 

### Análise de caminhos

Foi ajustado um modelo de caminhos, de forma a se comprovar o modelo teórico elencado sobre a influência do consumo de álcool em desfechos negativos da COVID-19, como a hospitalização e a COVID longa.

Na Figura 2, é possível observar que o uso de álcool não influenciou diretamente no aparecimento da COVID longa, por outro lado, seu consumo foi associado à hospitalização, sendo diretamente relacionado com a COVID longa. 

A análise de caminho revelou associações significativas entre diferentes variáveis estudadas. O uso de álcool demonstrou uma associação positiva com a hospitalização por COVID-19, em que, para cada aumento unitário na medida de uso de álcool, a chance de ser hospitalizado por COVID-19 aumentou em cerca de 7%. Por outro lado, a idade apresentou uma associação negativa com o uso de álcool, indicando que com o aumento na idade, a chance de usar álcool diminui em cerca de 1%. A escolaridade foi associada positivamente ao uso de álcool, mostrando um aumento de cerca de 11% na chance de uso de álcool à medida que o nível de escolaridade aumenta. A presença de DCNT no paciente acarreta um aumento de 11% na chance de hospitalização e 12% na chance de COVID de longa duração.

Da mesma forma, percebe-se que a idade dos participantes afetou no uso de álcool, com as maiores influências nas idades mais próximas da adultidade, bem como afetou diretamente a necessidade de hospitalização. Ainda, a escolaridade foi associada ao uso de álcool, conforme já mencionado.

**Figura 2:** Análise de caminhos do impacto do uso de bebidas alcoólicas nos desfechos da COVID-19

*OR = *Odds Ratio*; ^†^IC = Intervalo de Confiança; ^‡^DCNT = Doenças crônicas não transmissíveis

## Discussão

O perfil sociodemográfico da amostra estudada enfatizou as condições do padrão de consumo de álcool. Grande parte dos participantes não consumiram bebida alcoólica antes de contrair a COVID-19. Em outro estudo anterior, a maioria dos participantes (59,7%) indicou uma redução no consumo desde as medidas de bloqueio social^8^. 

A diminuição no uso de álcool pode ter sido impulsionada pelo acesso reduzido a bebidas alcoólicas e a diminuição da renda mensal para comprá-las. Muitos indivíduos perderam seus empregos durante o isolamento social no início da COVID-19, o que resultou em redução ou cessação da renda familiar e, em muitos casos, insegurança econômica e alimentar, conforme comprovado por outros estudos^22^ ^-^ ^23^. 

A instabilidade econômica gerada nesse período da COVID-19 resultou no aumento da pobreza e da vulnerabilidade social^24^. Portanto, tratar a COVID-19 como sindemia é considerá-la de forma integrada, considerando, também, os fatores sociais e econômicos da população afetada^25^.

O consumo de bebida alcoólica antes da COVID-19 esteve relacionado a pessoas do sexo masculino. O estudo realizado por inquérito nacional transversal na Eslovénia, utilizado para monitorar o número de bebidas alcoólicas consumidas durante a pandemia em comparação com o ano anterior à COVID-19, apresentou mudanças no consumo na primeira etapa da coleta para as faixas etárias adultas mais jovens, homens e pessoas com ensino superior. Isso pode ser explicado pela flexibilidade de medidas de comercialização, restaurantes, hotéis e centros comerciais no início da pesquisa^26^.

Em um outro estudo realizado em Berlim, cujo objetivo geral era gerar dados representativos sobre estresse relacionado à pandemia, estresse parental, saúde subjetiva e mental de pais e ocorrência de *Adverses Childhood Experiences* (ACEs), evidenciou-se que os pais se sentiram mais estressados pelo fechamento de escolas (55,9%), fechamento de creches (52,1%) e restrições a atividades externas (46,1%). Além disso, o estresse parenteral foi significativamente maior do que os níveis pré-COVID-19. Os pais dessa amostra relataram sintomas de ansiedade e depressão e, ainda, 37% dos pais relataram abuso de álcool ou substâncias no domicílio, ao longo da vida das crianças, sendo que, em 5,1%, os problemas aumentaram substancialmente (11,3%) durante a pandemia^27^. 

Este estudo evidenciou, pelo modelo de caminhos, que o consumo de bebida alcoólica não influenciou diretamente no aparecimento da COVID longa, mas que seu consumo foi associado à hospitalização, sendo este diretamente relacionado com a COVID longa. 

Nesta associação hierárquica, pode-se considerar que o uso do álcool é um fator de risco para o prolongamento da doença em indivíduos infectados pela COVID-19, assim como para um potencial agravamento dos casos, gerando mais hospitalizações^28^. 

O álcool tem efeito prejudicial no sistema imune, prejudica a barreira mucociliar do trato respiratório, reduz a resposta inata e a resistência ao vírus. Fato que aumenta a chance de complicações pulmonares após SARS-Cov-2^29^ e aumento da probabilidade de hospitalização^30^.

Neste estudo, as pessoas que ingeriram álcool antes da COVID-19 estavam mais susceptíveis à necessidade de hospitalização, seja em enfermaria ou na presença de gravidade do quadro, em UTI. Embora a carga viral seja um fator de risco importante, compreende-se que, isoladamente, níveis ou padrões de consumo de álcool aumentam o risco de consequências prejudiciais não apenas em dimensões físicas, mas também mentais e sociais para quem o consome^28^. 

Em relação às doenças crônicas analisadas, observou-se que a maioria dos participantes que ingeriram bebida alcoólica previamente apresentavam alguma doença preexistente. 

Num estudo realizado com uma amostra de 821 homens diagnosticados com transtorno por uso de substâncias, os quais foram internados em unidade de internação especializada em drogadição de um hospital público de Porto Alegre, Brasil, evidenciou-se que 305 eram usuários de álcool, 233 usuários de cocaína/crack e 283 homens que faziam uso de múltiplas substâncias (que incluíam álcool) e, destes, os usuários de álcool conferiram um número maior de fatores de risco para formas mais graves de COVID-19 em comparação com os demais grupos. O uso de álcool esteve relacionado às doenças metabólicas e hepáticas, além de prevalência de hipertensão (26,6% até 38%), cardiopatia (6,7% até 11,5%) e cirrose (4,4% até 7,9%)^28^_._

Cabe destacar que fragilidades existentes em termos de saúde física também podem refletir nas condições de risco à saúde geral. Assim, ponderando a prevalência de adultos e idosos que ingeriram álcool antes da COVID-19, existem fatores de risco para gravidade da COVID-19^29^, que representam maiores chances de hospitalização, os quais podem ser relacionados com a manifestação da COVID longa.

Foi apontado, em estudo de coorte online, com o objetivo de determinar os fatores sociodemográficos, que o estilo de vida bem como o histórico médico precedente à COVID-19 ou as características da infecção do SARS-CoV- 2 estiveram associadas à COVID longa. A mesma pesquisa ainda apresentou uma média de participantes que ingeriram bebida alcoólica pré-COVID-19 (4,19±5.71), sendo que, destes, 47,7% ainda relataram, no momento da pesquisa, sintomas de COVID longa^12^. Outra análise destacou que os adultos participantes com altas taxas de uso de álcool (27,3%) tiveram sintomas de COVID longa^31^ *.*

Ainda, as possíveis sequelas do consumo nas pessoas com vulnerabilidade devido a doenças subjacentes podem ter impactado consubstancialmente a qualidade de vida dos entrevistados, bem como viabilizaram uma repercussão negativa na sua saúde, pois as bebidas alcoólicas afetam direta ou indiretamente o sistema imunológico, conforme apontado por estudo prévio^32^.

Diante do exposto, enfatiza-se a importância de que estudos futuros considerem as especificidades socioeconômicas associadas ao consumo de álcool e o padrão de gravidade associado à COVID-19 e à COVID longa, a fim de ampliar as evidências e contribuir com estratégias de promoção e prevenção à saúde entre a população de adultos e, principalmente, de idosos, visto que os mesmos podem apresentar doenças crônicas diretamente associadas à gravidade da COVID longa. 

Refletir sobre o conceito de sindemia^2^ permite construir a dimensão entre as questões biológicas, para o desenvolvimento de uma doença, e questões sociais, que as favorecem, evidenciando, então, como esses fatores interagem entre si tanto em nível populacional como individual, para agravar a carga das doenças^33^.

As limitações do estudo referem-se, principalmente, ao viés das respostas, que podem ter sido influenciadas pela recordação dos participantes, familiares e/ou cuidadores, bem como ao fato de os dados serem autorrelatados ou até mesmo não respondidos. Isso pode induzir ao viés em nossa estimativa de pessoas que ingeriram bebida alcoólica antes da COVID-19. 

Entretanto, apesar das limitações citadas, é importante destacar que o estudo foi conduzido com dados de uma importante coorte realizada no estado do Paraná, a qual obteve um número expressivo de participantes e foi pioneira na utilização de tal método no estado durante o período de pandemia. Ainda, reforça-se que foram realizados diversos testes para minimizar o risco de viés de respostas e perda de dados que fossem relevantes para a compreensão do cenário epidemiológico relacionado à COVID-19 e COVID longa no estado do Paraná.

O estudo contribui para o avanço do conhecimento científico ao evidenciar a complexa interação entre a sindemia, o consumo de bebidas alcoólicas e a COVID-19, especialmente em adultos e idosos. Ao integrar múltiplas dimensões (biológica, social e comportamental), a pesquisa contribui para abordagens mais integradas e intersetoriais no planejamento de ações em saúde coletiva.

No campo da enfermagem, a pesquisa fortalece a compreensão da sindemia como um conceito essencial para qualificar a prática clínica, a vigilância em saúde e a formulação de intervenções educativas e preventivas. Além de destacar o papel estratégico da enfermagem na promoção da saúde e no cuidado integral em contextos complexos.

## Conclusão

Nesta avaliação transversal, ancorados em dados de uma coorte retrospectiva conduzida com adultos e idosos que tiveram COVID-19 no estado do Paraná, encontrou-se que o uso de álcool não influenciou diretamente no aparecimento da COVID longa, mas seu consumo foi associado à hospitalização, sendo diretamente relacionado com a COVID longa. Além disso, a faixa etária dos participantes afetou no consumo de álcool, com as maiores influências nas idades mais próximas da adultidade, bem como afetou diretamente a necessidade de hospitalização.

 Além disso, o uso de álcool foi mais associado às pessoas do sexo masculino e com maior escolaridade, mostrando um aumento de cerca de 11% na chance de uso de álcool à medida que o nível de escolaridade aumenta. A presença de DCNT no paciente acarreta um aumento de 11% na chance de hospitalização e 12% na chance de COVID de longa duração.

Portanto, este estudo recomenda medidas práticas, como protocolos de triagem para consumo de álcool em pacientes pós-COVID-19, especialmente na atenção primária, além disso, campanhas educacionais em saúde pública sobre os riscos do álcool em crises sanitárias, focando em grupos vulneráveis (como homens e indivíduos com maior escolaridade) e em capacitação profissional são importantes para integrar a pauta do álcool no manejo de pacientes com COVID-19 e COVID longa. 

## Declaração de Disponibilidade de Dados:

Os conjuntos de dados relacionados a este artigo estarão disponíveis mediante solicitação ao autor correspondente.

# Translation: Influencia del alcohol en el agravamiento de la COVID-19 y en la ocurrencia de COVID persistente^3^

**DOI:** 10.1590/1518-8345.7679.4812

**Authors:** Mirella Machado Ortiz Modesto, Natan Nascimento de Oliveira, Natan David Pereira, Wanessa Cristina Baccon, Lígia Carreira, Maria Aparecida Salci

**Affiliations:** 1 Universidade Estadual de Maringá, Maringá, PR, Brasil.; 2 Becaria (o) de la Coordenação de Aperfeiçoamento de Pessoal de Nível Superior (CAPES), Brasil.

**Keywords:** Consumo de Bebidas Alcoólicas, COVID-19, Sindémico, Síndrome Post Agudo de COVID-19, Adulto, Anciano.

## Abstract

**Objetivo:**

analizar el patrón de consumo de alcohol de adultos y personas mayores antes del desarrollo de la COVID-19 y la influencia del consumo de alcohol en los desenlaces y el agravamiento de la COVID persistente.

**Método:**

estudio transversal anclado en datos de una cohorte retrospectiva realizada con adultos y personas mayores que tuvieron COVID-19 y que consumieron alcohol antes de la infección por la enfermedad. Se utilizó un formulario electrónico estandarizado para la recolección de datos de la muestra y se ajustó un modelo de trayectorias, a fin de comprobar el modelo teórico sobre la influencia del consumo de alcohol en desenlaces negativos de la COVID-19.

**Resultados:**

muestra con 1.171 participantes que respondieron a la pregunta sobre alcohol. De estos, 408 (34,84%) informaron consumo de alcohol antes de la enfermedad. Predominó el sexo masculino, con edades más bajas, escolaridad elevada y con hijos mayores de 18 años. La presencia de Enfermedades Crónicas No Transmisibles conlleva un aumento del 11% en la probabilidad de hospitalización y del 12% en la probabilidad de COVID-19 persistente. La edad de los participantes influyó en el consumo de alcohol y afectó directamente la necesidad de hospitalización.

**Conclusión:**

es importante adoptar estrategias de intervención orientadas a la reducción del consumo de alcohol, sobre todo en contextos de sindemia, con el fin de mitigar los riesgos asociados.

## Destacados

**(1)** Quienes ingirieron alcohol antes de la COVID-19 tuvieron mayor riesgo de hospitalización. **(2)** El consumo de alcohol se relacionó con la hospitalización en la COVID persistente. **(3)** La mayoría de los participantes que consumían alcohol presentaban alguna enfermedad preexistente.

## Introducción

La COVID-19 desencadenó una inestimable crisis sanitaria, contribuyendo a la ampliación de las desigualdades sociales entre diversas poblaciones del mundo e instituyó diferentes modos de afrontar una nueva rutina social, debido a las alteraciones políticas, culturales y económicas^1^.

En virtud de ese hecho, diversos autores^2^ ^-^ ^4^ señalaron las características sindémicas de la COVID-19, pues además de haber producido repercusiones a nivel global, también generó efectos sinérgicos causados por la interacción de la COVID-19 con otras enfermedades ya existentes, que incrementaron la incidencia y potenciaron los efectos sociales^5^ ^-^ ^6^.

Se implementaron diversas intervenciones de salud pública durante la pandemia, incluyendo el distanciamiento social y la prohibición del funcionamiento de bares, restaurantes y locales nocturnos. Este comportamiento social fomentó un aumento considerable del consumo de alcohol y la adopción de comportamientos de abuso etílico en espacios privados/domicilio, sumado a vulnerabilidades preexistentes, lo que resultó en altos niveles de estrés, mayor tensión en las relaciones familiares y mayor riesgo de desarrollar trastornos mentales y diversos sufrimientos^7^ ^-^ ^9^.

En este contexto, es importante destacar que el consumo de alcohol se relaciona con factores sociales, comportamentales, psicológicos y económicos. Estos factores expresan el patrón de consumo de bebidas alcohólicas de la población brasileña y mundial^10^.

Además, el consumo crónico de alcohol perjudica el sistema inmunológico, volviendo al organismo susceptible a infecciones virales y bacterianas, con gran impacto en infecciones respiratorias graves, como la COVID-19^11^.

En un estudio realizado con 416 adultos de los Estados Unidos de América (EUA), 62,9% informó aumento del consumo de alcohol durante la pandemia, con 14,9% más de consumo intensivo (p<0,001) y un incremento de 65% en abuso de alcohol (p=0,0439). Además, se registró facilidad de acceso al alcohol (76%)^12^. Otra investigación, también en los EUA, reveló un aumento de 20% en el patrón de consumo de bebidas alcohólicas durante el período de la pandemia^13^.

Las personas que desarrollaron la COVID-19 o con antecedentes de probable infección también estuvieron sujetas a presentar síntomas a largo plazo, más conocidos como secuelas posagudas de la infección por SARS-CoV-2 (PASC) o “Long-COVID”. Los síntomas de COVID persistente son diversos e incluyen fatiga, disnea, confusión mental, problemas de memoria y concentración, dolor torácico y articular, y disfunción multiorgánica; pueden reaparecer tras la recuperación inicial de la fase aguda de la COVID-19 o persistir desde la primera ocurrencia de la enfermedad^14^.

La condición de COVID persistente ocurre, por lo general, tres meses después del inicio de la COVID-19, con síntomas que duran al menos dos meses. Actualmente no existen tratamientos comprobados para COVID persistente y sus síntomas no pueden explicarse por un diagnóstico alternativo^14^ ^-^ ^15^.

Se destaca que, en contextos afectados por la COVID-19, la interacción con otras enfermedades, principalmente Enfermedades Crónicas No Transmisibles (ECNT), impulsada por factores políticos, económicos y sociales, se caracterizó como sindemia^2^.

Así, producir nuevos conocimientos, profundizando en la comprensión de la vivencia del proceso de consumo de bebidas alcohólicas antes y después de la COVID-19, de los cambios ocurridos en el estilo/modo de vida individual y familiar durante este período, así como de las estrategias utilizadas para el mantenimiento de la salud física, la disminución de enfermedades y los daños a la salud, se torna imperativo para la ciencia^16^.

Ante este escenario, se pretende responder a la siguiente pregunta de investigación: ¿el consumo de bebidas alcohólicas, por adultos y personas mayores, antes de la COVID-19 agravó el cuadro de la enfermedad y ejerció influencia en la ocurrencia de la COVID persistente? En ese sentido, el objetivo del estudio fue analizar el patrón de consumo de alcohol de adultos y personas mayores que desarrollaron la COVID-19 y la influencia del consumo de alcohol en los desenlaces y el agravamiento de la COVID persistente.

## Método

### Diseño del estudio

Se trata de un estudio transversal retrospectivo, anclado en datos de una cohorte retrospectiva conducida con adultos y personas mayores que tuvieron COVID-19 en el estado de Paraná^17^. El estudio siguió las recomendaciones de *STrengthening the Reporting of OBservational studies in Epidemiology* (STROBE) en su construcción.

### Lugar

El presente estudio integró un proyecto mayor denominado “Cohorte COVID-19 Paraná/UEM”, desarrollado en la región Sur de Brasil, específicamente en el estado de Paraná, y utiliza el compartimiento de datos como estrategia metodológica. El objetivo principal del proyecto fue investigar las repercusiones a corto, mediano y largo plazo de la COVID-19 en adultos y personas mayores residentes en el estado de Paraná. La investigación se llevó a cabo en todo el estado, el cual abarca 399 municipios organizados en 22 Regionales de Salud y cuatro Macrorregionales: Este, Oeste, Norte y Noroeste. Hasta el 31 de diciembre de 2020, Paraná registró 413.412 casos confirmados de COVID-19 y 7.912 defunciones por la enfermedad^18^.

### Período

Los datos del proyecto se recolectaron entre el 11 de marzo de 2020 y el 31 de diciembre de 2020, tras un período mínimo de 12 meses a partir de la notificación o del alta hospitalaria de casos confirmados de COVID-19 por SARS-CoV-2, conforme a los registros en los sistemas de notificación. Sin embargo, la recolección de los datos de la muestra se inició en abril de 2021 y finalizó en marzo de 2022, con duración mínima de 50 minutos cada entrevista.

### Población

Participaron de este estudio: adultos, con edades entre 18 y 59 años completos, así como personas mayores a partir de 60 años, que fueron diagnosticados con COVID-19 o COVID persistente, y que residían en el estado de Paraná.

### Criterios de selección

Los criterios de inclusión para este estudio fueron: tener edad superior a 18 años; tener diagnóstico confirmado de COVID-19 o COVID persistente. Respecto a los criterios de exclusión, se removieron las observaciones en las que las respuestas a las preguntas “Antes de la COVID-19, ¿tomaba alguna bebida alcohólica?” y “¿COVID persistente?” eran nulas, así como se excluyeron gestantes, puérperas y participantes que fallecieron por cualquier motivo entre el período de la notificación y la aplicación del cuestionario.

### Definición de la muestra o participantes

A partir de las fichas de notificación compulsoria obligatoria del Síndrome Respiratoria Aguda Grave asociada al Coronavirus (SARS-CoV) en el Estado de Paraná, registradas en el Sistema de Vigilancia Epidemiológica de Síndrome Gripal (Sivep Gripe), sistema accesible para todos los núcleos de vigilancia epidemiológica para el monitoreo y control de los casos graves notificados, fueron seleccionados, por método de muestreo estratificado descrito en el estudio mayor^17^, 1.190 potenciales participantes, entre adultos y personas mayores diagnosticados con COVID-19 o COVID persistente y que recibieron atención en servicios de salud públicos y/o privados en el período entre el 11 de marzo de 2020 y el 31 de diciembre de 2020.

Tras la aplicación de los criterios de selección, el estudio finaliza con 1.171 observaciones, en las que los participantes se distribuyeron en tres subgrupos distintos: quienes recibieron atención ambulatoria 312 (41%), quienes fueron hospitalizados en salas de hospitalización 220 (29%) y quienes requirieron internación en UCI 231 (30%), tanto en el Sistema Único de Salud (SUS) como en instituciones privadas en el estado de Paraná.

### Variables del estudio

Se enumeraron como variables dependientes la ocurrencia de COVID persistente (no y sí), el uso de alcohol antes de la COVID-19 (no y sí) y la hospitalización para el tratamiento de la COVID-19 (no y sí). El desenlace primario fue la ocurrencia de COVID persistente, mientras que el alcohol y la hospitalización se consideraron desenlaces secundarios y mediadores de la COVID persistente.

Como variables independientes, se listaron características sociodemográficas y clínicas de los participantes, a saber: sexo (femenino y masculino), edad (media), grupo etario (adulto o persona mayor), raza/color (blanca y negro, pardo, amarillo e indígena), escolaridad (hasta 7 años de estudio y 8 años o más), situación conyugal (con pareja y sin pareja), número de hijos menores de 18 años (no y sí), enfermedad crónica (no y sí) y lugar de tratamiento de la COVID-19 aguda (ambulatorio, sala de hospitalización y UCI).

### Instrumentos utilizados para la recolección de información

Como fuente de datos para el estudio mayor, se utilizaron los registros de notificación del *Sistema Estadual Notifica COVID-19* (*Notifica COVID-19*) del estado de Paraná y, para los casos con internación hospitalaria (Enfermería y Unidad de Terapia Intensiva (UCI)), los participantes del estudio fueron identificados y seleccionados a partir de las fichas de notificación compulsoria de la Síndrome Respiratoria Aguda Grave (SRAG) asociada al SARS-CoV-2, insertadas en el Sistema de Vigilancia Epidemiológica de Síndrome Gripal (SIVEP-Gripe).

El instrumento utilizado para la recolección de datos de la investigación fue un formulario electrónico estandarizado, aplicado por los miembros (enfermeros y estudiantes de posgrado del área de la salud) integrantes del proyecto “Cohorte COVID-19 Paraná/UEM”. El instrumento está compuesto por cuatro bloques temáticos principales, clasificados de la siguiente forma: (I) Identificación inicial del(a) participante y del/de la entrevistador(a); (II) Historial de la Cohorte (subdividido en el bloque (II.1) Signos y síntomas de los síndromes COVID y pos-COVID; (II.2) Historial de la COVID-19 y pos-COVID-19; (II.3) Historial personal); (III) Caracterización sociodemográfica, laboral y económica/financiera del(a) participante y (IV) Información final de la entrevista.

La pregunta sobre el patrón de consumo de bebidas alcohólicas antes de la COVID-19 forma parte del bloque II.3 Historial personal. Se preguntó al participante si bebe/bebía alguna bebida alcohólica y, para los casos de respuestas positivas, se verificó cuántos días por semana se realizaba ese consumo (1 a 7 días), cuáles eran los tipos de bebida (cerveza; vino; martini/vermú; *cachaça*/vodka/whisky) y la cantidad aproximada diaria, en mililitros (ml).

Se emplearon, como parámetro de las unidades alcohólicas brasileñas, los valores de la OMS vigentes en el período^19^, los cuales fueron actualizados más tarde, manteniendo la misma fórmula de cálculo, con base en dosificaciones ligeramente diferentes. Para la OMS, una dosis estándar contiene 10 gramos (g), o 12,7 mililitros (mL) de etanol puro. Esa dosis equivale a 285 mL de una cerveza común, 100 mL de vino o 30 mL de destilados^20^.

### Recolección de datos

Las entrevistas se realizaron por medio de llamadas telefónicas grabadas, con duración mínima de 50 minutos, utilizando un formulario electrónico como guía. La recolección de datos fue conducida por enfermeros y estudiantes de posgrado del área de la salud, quienes recibieron capacitación de al menos 20 horas para la realización de la investigación.

Tras la recolección de datos, el Consentimiento Informado (CI) fue enviado a los participantes en formato PDF, por medio de plataformas en línea o por correo electrónico. Se optó por realizar entrevistas por teléfono o en línea para superar limitaciones geográficas, permitiendo la participación de pacientes en todas las regiones del estado de Paraná, y, también, para seguir las recomendaciones de distanciamiento social.

Los datos recolectados se almacenaron en una base de datos que permitía solo un envío del formulario por IP (Protocolo de Internet), con el fin de garantizar la seguridad de la información obtenida.

La información personal y/o sensible que permitía la identificación y el contacto con los participantes se obtuvo previamente de las fichas de registro individual, combinando tres bases de datos: la base de vigilancia epidemiológica de la COVID-19 de Brasil, la base del Sistema de Vigilancia Epidemiológica de Síndrome Gripal (Sivep Gripe) y el Sistema Estadual de Notificación de la COVID-19 de Paraná, conocido como *Notifica COVID-19 Paraná*. Las bases 1 y 2 abarcan casos de internación por Síndrome Respiratoria Aguda Grave (SRAG) debido a la COVID-19 (casos graves y moderados), mientras que la base 3 incluye casos ambulatorios o leves de la enfermedad.

### Tratamiento y análisis de los datos

Los datos se tabularon en hojas de cálculo y se analizaron mediante el *software* R, versión 4.3.0. Se realizó un análisis descriptivo de las variables, por medio de la estimación de frecuencias absolutas y relativas de las variables categóricas, además de media y desviación estándar de las variables numéricas. Las pruebas chi-cuadrado de Pearson y la prueba de Mann-Whitney se utilizaron para identificar diferencias entre grupos. Para comprender las relaciones complejas entre el uso de alcohol y la COVID-19, se ajustaron Modelos de Ecuaciones Estructurales (MEE)^21^, específicamente mediante análisis de trayectorias (*Path Analysis*). Esta técnica multivariada se empleó porque permite probar modelos teóricos hipotetizados al mismo tiempo que estima las relaciones directas e indirectas entre múltiples variables. Este enfoque es particularmente adecuado para investigar fenómenos multifactoriales, como las interacciones propuestas entre COVID-19, uso de alcohol y determinantes sociales de la salud. Se consideraron variables exógenas (solo causantes), es decir, aquellas que influyen en otras variables dentro del modelo sin ser influidas por ninguna de ellas en el alcance de este estudio; en este grupo se incluyeron Edad, Sexo, Estado civil y Escolaridad. Ya las variables endógenas (causadas por otras variables/mediadoras) son aquellas que están influenciadas por una o más variables dentro del modelo. Cada variable endógena se modeló como un desenlace para una o más variables predictoras, a saber: Uso de alcohol antes de la COVID-19, Enfermedad Crónica No Transmisible (ECNT), Hospitalización para el tratamiento de la COVID-19 y, por último, COVID persistente, esta última representando el desenlace principal que fue modelado, estando influenciada por todas las variables anteriores, directa y/o indirectamente (Figura 1). Este modelo tuvo en cuenta el concepto de la COVID-19 como sindemia^2^ de los trastornos relacionados con el uso de alcohol y los determinantes sociales de la salud. Las relaciones propuestas fueron, entonces, cuantificadas por medio de los coeficientes estimados, en forma de *Odds Ratios*. Esta técnica de modelización por ecuaciones estructurales se basó en covarianza (CB-SEM) y estructura *logit*, utilizando el paquete *lavaan* en R. Como criterios de análisis, todas las variables pasaron por la verificación de multicolinealidad (VIF), chi-cuadrado, *Comparative Fit Index* (CFI), *Loglikelihood y Root Mean Square Error of Approximation* (RMSEA).

**Figura 1:** Modelo teórico de la influencia del alcohol sobre los desenlaces de la COVID-19

*ECNT = Enfermedades Crónicas No Transmisibles

### Aspectos éticos

Se observaron los preceptos éticos de las Resoluciones n.º 466/2012 y n.º 510/2016, del Consejo Nacional de Salud (CNS) (Brasil, 2012). El proyecto de investigación “Cohorte COVID-19 Paraná/UEM” cuenta con la aprobación del Comité Permanente de Ética en Investigación con Seres Humanos (COPEP) de la *Universidade Estadual de Maringá* (UEM), con dictamen n.º 4.165.272/2020 y con Certificado de Presentación para Apreciación Ética (CAAE) n.º 34787020.0.0000.0104, emitido el 21 de julio de 2020.

Se informó a los participantes involucrados en la recolección de datos sobre los objetivos y el método utilizado en la investigación, así como sobre la participación voluntaria, la disponibilidad y la voluntad de participar. Se garantizó el derecho de acceso a los datos.

El consentimiento libre e informado y la autorización para la grabación de las entrevistas se obtuvieron oralmente en el momento del contacto telefónico con los entrevistados. A continuación, el CI se enviaba a los participantes por medios electrónicos o por correo.

## Resultados

Se seleccionaron 1.171 participantes en el estudio que respondieron a las preguntas relativas al uso de alcohol. De estos, 763 (65,16%) indicaron no utilizar alcohol previamente a la COVID-19, mientras que 408 (34,84%) informaron consumo de alcohol antes de la enfermedad (Tabla 1).

**Tabla 1:** Características de los participantes del estudio que informaron el uso de alcohol antes de la infección por COVID-19. Maringá, PR, Brasil, 2024

Variables	Uso de alcohol antes de la COVID-19			
	No (N = 763)	Sí (N = 408)	Prueba	valor-p
**Sexo**			QQ*	<0,001
Femenino	440 (58%)	121 (30%)		
Masculino	323 (42%)	287 (70%)		
**Edad**			KW^†^	<0,001
Media (Desviación-estándar)	55,3 (16.0)	50,5 (15.4)		
**Grupo etario**			QQ*	<0,001
Adulto	381 (50%)	260 (64%)		
Persona mayor	382 (50%)	148 (36%)		
**Raza/Color**			QQ*	0,990
Blanca	494 (65%)	264 (65%)		
Negra/Parda/Amarilla/Indígena	269 (35%)	144 (35%)		
**Escolaridad**			QQ*	<0,001
Hasta 7 años de estudio	310 (41%)	104 (25%)		
8 años o más de estudio	453 (59%)	304 (75%)		
**Situación conyugal**			QQ*	0,844
Con pareja	513 (67%)	272 (67%)		
Sin pareja	250 (33%)	136 (33%)		
**Hijos menores de 18 años**			QQ*	0,009
No	655 (86%)	326 (80%)		
Sí	108 (14%)	82 (20%)		
**Enfermedad crónica**			QQ*	0,055
No	258 (34%)	161 (39%)		
Sí	505 (66%)	247 (61%)		
**Lugar de tratamiento de la COVID aguda**			QQ*	0,386
Ambulatorio	312 (41%)	150 (37%)		
Sala de hospitalización	220 (29%)	125 (31%)		
UCI	231 (30%)	133 (33%)		
**COVID persistente**			QQ*	0,336
No	251 (33%)	123 (30%)		
Sí	512 (67%)	285 (70%)		

*QQ = Prueba de chi-cuadrado de Pearson; ^†^KW = Prueba de Kruskal-Wallis

Entre los participantes que informaron consumo de alcohol, se observa mayor prevalencia del sexo masculino, con edades más bajas, escolaridad elevada y con hijos mayores de 18 años.

### Análisis de trayectorias

Se ajustó un modelo de trayectorias, con el fin de comprobar el modelo teórico propuesto sobre la influencia del consumo de alcohol en desenlaces negativos de la COVID-19, como la hospitalización y la COVID persistente.

En la Figura 2, es posible observar que el uso de alcohol no influyó directamente en la aparición de la COVID persistente; por otro lado, su consumo se asoció a la hospitalización, estando directamente relacionado con la COVID persistente.

El análisis de trayectorias reveló asociaciones significativas entre diferentes variables estudiadas. El uso de alcohol mostró una asociación positiva con la hospitalización por COVID-19, en la que, por cada aumento unitario en la medida de uso de alcohol, la probabilidad de ser hospitalizado por COVID-19 aumentó en cerca de 7%. Por otro lado, la edad presentó una asociación negativa con el uso de alcohol, indicando que con el aumento de la edad, la probabilidad de usar alcohol disminuye en cerca de 1%. La escolaridad se asoció positivamente con el uso de alcohol, mostrando un aumento de cerca de 11% en la probabilidad de uso de alcohol a medida que el nivel de escolaridad aumenta. La presencia de ECNT en el paciente conlleva un aumento de 11% en la probabilidad de hospitalización y de 12% en la probabilidad de COVID persistente.

De la misma forma, se observa que la edad de los participantes afectó el uso de alcohol, con mayores influencias en las edades más próximas a la adultez, así como afectó directamente la necesidad de hospitalización. Asimismo, la escolaridad se asoció al uso de alcohol, como ya se mencionó.

**Figura 2:** Análisis de trayectorias del impacto del uso de bebidas alcohólicas en los desenlaces de la COVID-19

*OR = *Odds Ratio*; ^†^IC = Intervalo de Confianza; ^‡^ECNT = Enfermedades crónicas no transmisibles

## Discusión

El perfil sociodemográfico de la muestra estudiada enfatizó las condiciones del patrón de consumo de alcohol. Gran parte de los participantes no consumió bebidas alcohólicas antes de contraer la COVID-19. En otro estudio anterior, la mayoría de los participantes (59,7%) indicó una reducción en el consumo desde las medidas de confinamiento social^8^.

La disminución en el uso de alcohol puede haber sido impulsada por el acceso reducido a bebidas alcohólicas y la disminución del ingreso mensual para comprarlas. Muchas personas perdieron sus empleos durante el aislamiento social al inicio de la COVID-19, lo que resultó en reducción o cese del ingreso familiar y, en muchos casos, inseguridad económica y alimentaria, como fue demostrado por otros estudios^22^ ^-^ ^23^.

La inestabilidad económica generada en ese período de la COVID-19 resultó en el aumento de la pobreza y de la vulnerabilidad social^24^. Por lo tanto, tratar la COVID-19 como sindemia implica considerarla de forma integrada, teniendo en cuenta también los factores sociales y económicos de la población afectada^25^.

El consumo de bebidas alcohólicas antes de la COVID-19 estuvo relacionado con personas del sexo masculino. El estudio realizado por encuesta nacional transversal en Eslovenia, utilizado para monitorear el número de bebidas alcohólicas consumidas durante la pandemia en comparación con el año anterior a la COVID-19, presentó cambios en el consumo en la primera etapa de la recolección para los grupos etarios adultos más jóvenes, hombres y personas con educación superior. Esto puede explicarse por la flexibilización de medidas en el comercio, restaurantes, hoteles y centros comerciales al inicio de la investigación^26^.

En otro estudio realizado en Berlín, cuyo objetivo general fue generar datos representativos sobre estrés relacionado con la pandemia, estrés parental, salud subjetiva y mental de padres y ocurrencia de *Adverse Childhood Experiences* (ACEs), se evidenció que los padres se sintieron más estresados por el cierre de escuelas (55,9%), cierre de guarderías (52,1%) y restricciones a actividades externas (46,1%). Además, el estrés parental fue significativamente mayor que los niveles pre-COVID-19. Los padres de esa muestra informaron síntomas de ansiedad y depresión y, asimismo, 37% de los padres informó abuso de alcohol o sustancias en el domicilio a lo largo de la vida de los niños, siendo que, en 5,1%, los problemas aumentaron sustancialmente (11,3%) durante la pandemia^27^.

Este estudio evidenció, por el modelo de trayectorias, que el consumo de bebidas alcohólicas no influyó directamente en la aparición de la COVID persistente, pero que su consumo se asoció a la hospitalización, estando ésta directamente relacionada con la COVID persistente.

En esta asociación jerárquica, puede considerarse que el uso de alcohol es un factor de riesgo para el prolongamiento de la enfermedad en individuos infectados por la COVID-19, así como para un potencial agravamiento de los casos, generando más hospitalizaciones^28^.

El alcohol tiene un efecto perjudicial sobre el sistema inmune, deteriora la barrera mucociliar del tracto respiratorio, reduce la respuesta innata y la resistencia al virus. Hecho que aumenta la probabilidad de complicaciones pulmonares tras el SARS-CoV-2^29^ y el incremento de la probabilidad de hospitalización^30^.

En este estudio, las personas que ingirieron alcohol antes de la COVID-19 estaban más susceptibles a la necesidad de hospitalización, ya sea en sala de hospitalización o, en presencia de gravedad del cuadro, en UCI. Aunque la carga viral sea un factor de riesgo importante, se comprende que, aisladamente, niveles o patrones de consumo de alcohol aumentan el riesgo de consecuencias perjudiciales no solo en dimensiones físicas, sino también mentales y sociales para quien lo consume^28^.

Con relación a las enfermedades crónicas analizadas, se observó que la mayoría de los participantes que ingirieron bebidas alcohólicas previamente presentaban alguna enfermedad preexistente.

En un estudio realizado con una muestra de 821 hombres diagnosticados con trastorno por uso de sustancias, internados en una unidad de hospitalización especializada en drogadicción de un hospital público de Porto Alegre, Brasil, se evidenció que 305 eran usuarios de alcohol, 233 usuarios de cocaína/crack y 283 hombres que hacían uso de múltiples sustancias (que incluían alcohol) y, de estos, los usuarios de alcohol presentaron un mayor número de factores de riesgo para formas más graves de COVID-19 en comparación con los demás grupos. El uso de alcohol estuvo relacionado con enfermedades metabólicas y hepáticas, además de la prevalencia de hipertensión (26,6% hasta 38%), cardiopatía (6,7% hasta 11,5%) y cirrosis (4,4% hasta 7,9%)^28^.

Cabe destacar que las fragilidades existentes en términos de salud física también pueden reflejarse en condiciones de riesgo para la salud general. Así, considerando la prevalencia de adultos y personas mayores que ingirieron alcohol antes de la COVID-19, existen factores de riesgo para la gravedad de la COVID-19^29^, que representan mayores probabilidades de hospitalización, los cuales pueden estar relacionados con la manifestación de la COVID persistente.

Se señaló, en un estudio de cohorte en línea, con el objetivo de determinar los factores sociodemográficos, que el estilo de vida, así como los antecedentes médicos previos a la COVID-19 o las características de la infección por SARS-CoV-2, estuvieron asociados a la COVID persistente. La misma investigación además presentó un promedio de participantes que ingirieron bebidas alcohólicas pre-COVID-19 (4,19±5,71), y, de estos, 47,7% aún informó, en el momento de la investigación, síntomas de COVID persistente^12^. Otro análisis destacó que los adultos participantes con altas tasas de uso de alcohol (27,3%) presentaron síntomas de COVID persistente^31^.

Asimismo, las posibles secuelas del consumo en personas con vulnerabilidad debido a enfermedades subyacentes pueden haber impactado de manera consubstancial la calidad de vida de los entrevistados, así como posibilitado una repercusión negativa en su salud, pues las bebidas alcohólicas afectan directa o indirectamente el sistema inmunológico, según lo señalado por un estudio previo^32^.

Ante lo expuesto, se enfatiza la importancia de que estudios futuros consideren las especificidades socioeconómicas asociadas al consumo de alcohol y el patrón de gravedad asociado a la COVID-19 y a la COVID persistente, con el fin de ampliar la evidencia y contribuir con estrategias de promoción y prevención de la salud entre la población adulta y, principalmente, las personas mayores, dado que estas pueden presentar enfermedades crónicas directamente asociadas a la gravedad de la COVID persistente.

Reflexionar sobre el concepto de sindemia^2^ permite construir la articulación entre las cuestiones biológicas para el desarrollo de una enfermedad y las cuestiones sociales que la favorecen, evidenciando cómo estos factores interactúan entre sí tanto a nivel poblacional como individual para agravar la carga de las enfermedades^33^.

Las limitaciones del estudio se refieren, principalmente, al sesgo de las respuestas, que pueden haber sido influenciadas por el recuerdo de los participantes, familiares y/o cuidadores, así como al hecho de que los datos sean autorreportados o incluso no respondidos. Esto puede inducir sesgo en nuestra estimación de personas que ingirieron bebidas alcohólicas antes de la COVID-19.

Sin embargo, a pesar de las limitaciones citadas, es importante destacar que el estudio fue conducido con datos de una importante cohorte realizada en el estado de Paraná, la cual obtuvo un número expresivo de participantes y fue pionera en la utilización de tal método en el estado durante el período de pandemia. Asimismo, se refuerza que se realizaron diversas pruebas para minimizar el riesgo de sesgo de respuestas y la pérdida de datos relevantes para la comprensión del escenario epidemiológico relacionado con la COVID-19 y la COVID persistente en el estado de Paraná.

El estudio contribuye al avance del conocimiento científico al evidenciar la compleja interacción entre la sindemia, el consumo de bebidas alcohólicas y la COVID-19, especialmente en adultos y personas mayores. Al integrar múltiples dimensiones (biológica, social y comportamental), la investigación contribuye a enfoques más integrados e intersectoriales en la planificación de acciones en salud colectiva.

En el campo de la enfermería, la investigación fortalece la comprensión de la sindemia como un concepto esencial para cualificar la práctica clínica, la vigilancia en salud y la formulación de intervenciones educativas y preventivas. Además de destacar el papel estratégico de la enfermería en la promoción de la salud y en el cuidado integral en contextos complejos.

## Conclusión

En esta evaluación transversal, anclados en datos de una cohorte retrospectiva conducida con adultos y personas mayores que tuvieron COVID-19 en el estado de Paraná, se encontró que el uso de alcohol no influyó directamente en la aparición de la COVID persistente, pero su consumo se asoció a la hospitalización, estando directamente relacionado con la COVID persistente. Además, el grupo etario de los participantes afectó el consumo de alcohol, con mayores influencias en las edades más próximas a la adultez, así como afectó directamente la necesidad de hospitalización.

Además, el uso de alcohol estuvo más asociado a personas del sexo masculino y con mayor escolaridad, mostrando un aumento de cerca de 11% en la probabilidad de uso de alcohol a medida que el nivel de escolaridad aumenta. La presencia de ECNT en el paciente conlleva un aumento de 11% en la probabilidad de hospitalización y de 12% en la probabilidad de COVID persistente.

Por lo tanto, este estudio recomienda medidas prácticas, como protocolos de tamizaje para consumo de alcohol en pacientes pos-COVID-19, especialmente en la atención primaria; además, son importantes campañas educativas de salud pública sobre los riesgos del alcohol en crisis sanitarias, con foco en grupos vulnerables (como hombres e individuos con mayor escolaridad) y en la capacitación profesional, para integrar la temática del alcohol en el manejo de pacientes con COVID-19 y COVID persistente.
